# Clinical application of an Asian Screening Array-based preimplantation genetic testing workflow for various genetic disorders

**DOI:** 10.1186/s13023-026-04411-5

**Published:** 2026-06-08

**Authors:** Cuiting Peng, Jun Ren, Fan Zhou, Han Chen, Hong Yang, Yutong Li, Yuezhi Keqie, Xu Zhao, Zhushu Liu, Ting Hu, Xuemei Zhang, Taoli Ding, Ji Yang, Shan Luo, Wei Fan, Xinlian Chen, Shanling Liu

**Affiliations:** 1 Department of Medical Genetics Center, West China Second University Hospital, Sichuan University, No. 20, Section 3, Renmin South Road, Chengdu, China; 2 Key Laboratory of Birth Defects and Related Diseases of Women and Children, Sichuan University, Ministry of Education, Chengdu, China; 3 Yikon Genomics, Suzhou, China; 4 Department of Reproductive Medicine, West China Second University Hospital, Sichuan University, Chengdu, China

**Keywords:** Preimplantation genetic testing, Asian Screening Array, Monogenic disorder, Microdeletion/Microduplication, Haplotyping analysis

## Abstract

**Background:**

Preimplantation genetic testing for monogenic disorders (PGT-M) represents a critical clinical strategy for preventing the transmission of hereditary diseases from carriers to offspring, with its diagnostic efficacy heavily dependent on the accuracy and coverage of detection platforms. Genome-wide SNP array such as the Asian Screening Array (ASA), has demonstrated favorable performance in haplotype analysis for PGT-M. Here, we systematically evaluated the efficiency of the Asian Screening Array-based PGT workflow in PGT application for different genetic disorders.

**Methods:**

We conducted a retrospective analysis by reviewing 377 pedigrees underwent PGT-M preclinical work-up and 367 PGT-M clinical cycles (1677 embryos in total) based on the Asian Screening Array. We established detection strategies combining ASA haplotyping analysis and individualized direct mutation detection based on different genetic patterns. Long-read sequencing or single-sperm haplotyping strategy was applied for families with de novo pathogenic variants or lacking family member samples. Individualized direct mutation detection methods such as Gap-PCR, RP-PCR, PCR-RFLP were adopted for different cases.

**Results:**

Results indicated the clinical validity of our ASA-based PGT workflow, integrating linkage analysis, direct mutation detection, and chromosomal CNV screening. The number of SNPs available for linkage analysis in the upstream and downstream regions of target genes/regions is sufficient for most of the cases. Individualized direct mutation detection methods for different cases also validated the ASA haplotyping results. In haplotype analysis, the method based on long-read sequencing is more effective than single-sperm haplotyping strategy. Detailed haplotyping strategy for families with microdeletions and tandem duplications was established based on ASA. A total of 636 embryos were ultimately deemed suitable for transfer after undergoing linkage analysis and CNV detection.

**Conclusion:**

This study validated the feasibility and superiority of ASA-based approach in PGT, also provided a standardized and reliable technical solution for the clinical prevention of different genetic disorders.

**Supplementary Information:**

The online version contains supplementary material available at 10.1186/s13023-026-04411-5.

## Introduction

Preimplantation Genetic Testing (PGT) is an innovative approach designed to screen for and diagnose genetic abnormalities in early embryos prior to transfer [1, 2]. It not only provides a reliable avenue for couples at high risk of genetic disorders to have healthy children but also optimizes the outcomes of assisted reproductive therapies [3]. With the increasing global prevalence of genetic disorders, the demand for accurate and efficient PGT methodologies has become increasingly urgent [4].

With the continuous development and innovation of detection technologies, such as next generation sequencing (NGS), single-nucleotide polymorphism (SNP) array, long-read sequencing, the detection efficiency and application scope of PGT have been significantly enhanced [5, 6]. As for preimplantation genetic testing for monogenic defects (PGT-M), haplotyping analysis combined with direct mutation detection has proven to be an effective strategy to improve test accuracy [7]. Genome-wide SNP array represented by Karyomapping and the Asian Screening Array, has demonstrated favorable performance in haplotype analysis for PGT-M [8–10]. The Asian Screening Array (ASA) integrates high-density SNP loci with enhanced coverage of Asian-specific pathogenic variants, thus serving as a specialized array for East and Southeast Asian populations in linkage analysis and detection of population-specific genetic variants [11].

In recent years, more and more integrated PGT detection protocols which achieves simultaneous SNP linkage analysis, chromosomal aneuploidy screening (PGT-A), and chromosomal structural rearrangement detection (PGT-SR), has been extensively developed and applied [12–14]. However, it still suffers from drawbacks such as high costs, complexity of data analysis and limited detection efficacy in low-coverage regions. Thus, despite the requirement for an additional PGT-A assay, the SNP array-based linkage analysis protocol still retains considerable clinical utility, owing to its high accuracy in monogenic disorder detection and controllable costs. Moreover, the integration of ASA with advanced technologies such as long-read sequencing and single sperm haplotyping has opened new avenues for addressing complex scenarios in PGT, such as de novo pathogenic variants and lack of family member samples [15–17].

Long-read sequencing represented by Oxford nanopore sequencing technologies (ONT, UK) could generates ultra-long genomic reads and thus holds considerable potential for precise breakpoint detection and direct haplotype phasing [18, 19]. Meanwhile, the adaptive sampling model of nanopore sequencing can enrich regions of interest by rejecting off-target regions, thereby reducing the generation of redundant data and lowering the detection costs [20]. Based on the haplotype analysis results derived from single sperm or long-read sequencing, combined with the ASA array results of healthy spouses and embryos, the presence of risk haplotypes in embryos can be determined [16, 21].

The purpose of this study is to evaluate the efficiency of ASA-based PGT-M approach using a large clinical cohort, including 377 pedigrees and 1677 embryos with various monogenic diseases or small pathogenic CNVs. We established individualized detection strategy based on pedigree genetic patterns. The performance of the ASA combined with different mutation detection methods was comprehensively evaluated from both technical (detection accuracy and specificity) and clinical (embryo detection efficiency) perspectives. This study aims to validate the feasibility and superiority of ASA-based approach in PGT, providing a standardized, efficient, and reliable technical solution for the clinical prevention of different genetic disorders.

## Materials and methods

### Patients inclusion

This study included all the pedigrees underwent PGT-M preclinical (377 families) and clinical work-up (367 cycles) based on Asian Screening Array at West China Second University Hospital, Sichuan University, from August 2022 to July 2025. The pedigree members including prospective parents and close relatives with known disease status for linkage analysis. Pedigrees with *de novo* pathogenic variant(s) or lack of positive family members were also included. The present study was approved by the Ethics Committee of West China Second University Hospital of Sichuan University and performed in accordance with the Declaration of Helsinki. Informed consent was obtained from all the participants included in the study.

### Sample collection

In preclinical work-up stage, sample types include peripheral blood samples, fetal tissue samples, amniotic fluid cell samples or single sperm cells et al. Genomic DNA were extracted from those samples according to the manufacturer’s instructions (QIGEN, QIAamp DNA Micro Kit). For long-read sequencing, high-molecular weight genomic DNA were extracted and purified using QIAGEN Gentra Puregene Blood Kit. In PGT-M clinial work-up stage, Ovarian stimulation, in vitro fertilization and trophectoderm (TE) biopsy were conducted in the reproductive medical center according to the standard protocol [22, 23]. Whole-genome amplification (WGA) were conducted for biopsied TE cells or single sperm cells using the Multiple Displacement Amplification method (MDA, QIAGEN) or the multiple annealing and looping-based amplification cycles (MALBAC, Yikon genomics) [24]. Whole genome products were purified using DNA Clean-up Kit (CWBIO).

### SNPs detection and linkage analysis based on Asian screening array

For linkage analysis, SNPs were detected based on Infinium Asian Screening Array. Briefly, DNA samples were hybridized to Infinium Asian Screening Array-24 v1.0 BeadChips for extension and staining after amplified and fragmented. Then, BeadChips were scanned and imaged by iScan System (Illumina). Data was analyzed and SNPs or genotype calling was conducted on ChromGo (Yikon Genomics). Informative SNPs which were heterozygous in the affected/carrier parent and homozygous in the spouse were selected for linkage analysis. At least three informative SNPs proximal and distal to the region of interest were recommended for linkage analysis. Particularly, SNPs inside the microdeletion/microduplication region may provide useful information in some cases. For affected male participants with *de novo* pathogenic variant, single-sperm cell samples could be applied as phasing references to directly distinguish haplotypes.

### Long read sequencing and direct haplotype phasing by Oxford nanopore technology

For cases lacking of phasing references, direct haplotype phasing by long-read sequencing was recommended. The extracted high-molecular-weight genomic DNA of the affected/carrier parent was applied to sequence on GridION (Oxford Nanopore Technologies). The DNA sequencing library was prepared as described in our previous study [16]. The prepared DNA libraries were sequenced under adaptive sampling model to enrich regions of interest, usually 10 Mb flanking the target genes or regions, by rejecting off-target regions with no additional library preparation. Data processing and analysis workflow referred to our previous study. Usually, A depth of 30× is required for direct haplotye phasing. The mean quality score of base-calling and frequency of nucleotide in reads was also evaluated. The high-risk haplotype linked with pathogenic variant could be directly distinguished after data processing.

### Sanger sequencing validation

Targeted PCR amplification and Sanger sequencing were conducted on pedigree members and embryos with point mutations as well as small insertions, deletions, and duplications (indels). Specific primers were designed to amplify targeted segments containing mutation sites (Primer 5.0 software) and synthesized (TsingkeBiotechnologyCo., Ltd.). PCR amplifications were performed on a 96 Well Thermal Cycler Veriti DX (Life Technologies) with suitable PCR conditions. Subsequent Sanger sequencing was performed and data was analyzed using ChromasPro software.

### Repeat-primed PCR and fragment length analysis for repeat expansion disorders

For cases with abnormal expansion of trinucleotide repeats in *HTT*,* ATXN1*, *ATXN2*, *ATXN3* and *FMR1* genes in this study, Repeat-Primed PCR (RP-PCR) combined with downstream size separation and analysis was conducted for direct expansion detection [25, 26]. Briefly, a fluorescently labeled upstream primer, a universal primer and a tail primer that includes repeat sequences were used to amplify repeats of varying sizes by PCR. Then, the amplified DNA fragments were separated and analyzed using capillary electrophoresis on ABI 3500 Dx Generic Analyzer. Data was viewed and processed on GeneMapper 6 to calculate the numbers of trinucleotide repeats for each sample.

### Gap-PCR validation for common large deletional mutations of α-globin gene cluster

For deletional alpha-thalassemia cases such as the Southeast Asian type (--^SEA^), the Thai type (--^THAI^), -α^3.7^ and -α^4.2^, Gap-PCR analysis was needed for direct mutation detection [27, 28]. In our study, 8 pairs of primer named P1-F/R P2-F/R to P8-F/R flanking or within the deletion region were applied for gap-PCR. Thus, embryos carrying deletion-type mutations could be distinguished from normal or carrier embryos via gap-PCR combined with agarose gel electrophoresis.

### PCR-restriction fragment length polymorphism (PCR-RFLP) to identify homozygous deletion of exon 7 or 8 in SMN1

Owing to the presence of the *SMN2* gene, which is highly homologous to the *SMN1* gene, accurate diagnosis of exon 7/8 deletions in the *SMN1* gene requires first conducting differential diagnosis between the *SMN1* and *SMN2* genes. For the differentiation of exon 7 between the two genes, a specific primer pair (G1-F/R) was used to introduce a mismatched site into exon 7 of the *SMN2* gene through PCR amplification, which resulted in the formation of a specific DraI restriction enzyme site in the PCR product. As for exon 8, direct PCR amplification was performed using the G2-F/R primer pair to amplify the inherent DdeI restriction enzyme site within exon 8 of the *SMN2* gene. Subsequent restriction enzyme digestion of the product enabled the differentiation of exon 7/8 between *SMN1* and *SMN2*. A high-resolution agarose (MetaPhor Agrose, Lonza Bioscience) was adopted for agarose gel electrophoresis.

### Copy number variation (CNVs) analysis

In our study, embryos were performed low-depth whole-genome sequencing on Illumina platform to fulfill prospective chromosome analysis. DNA sequencing libraries of both MALBAC and MDA products were prepared and sequenced with no modifications as previously described [29]. Data analysis was conducted on the localized platform of ChromGO (Yikon Genomics). For participants with positive results from initial linkage analysis and direct mutation analysis, subsequent copy number variation analysis can be omitted.

## Results

### Overall information of PGT-M preclinical work-up

**Fig. 1:**
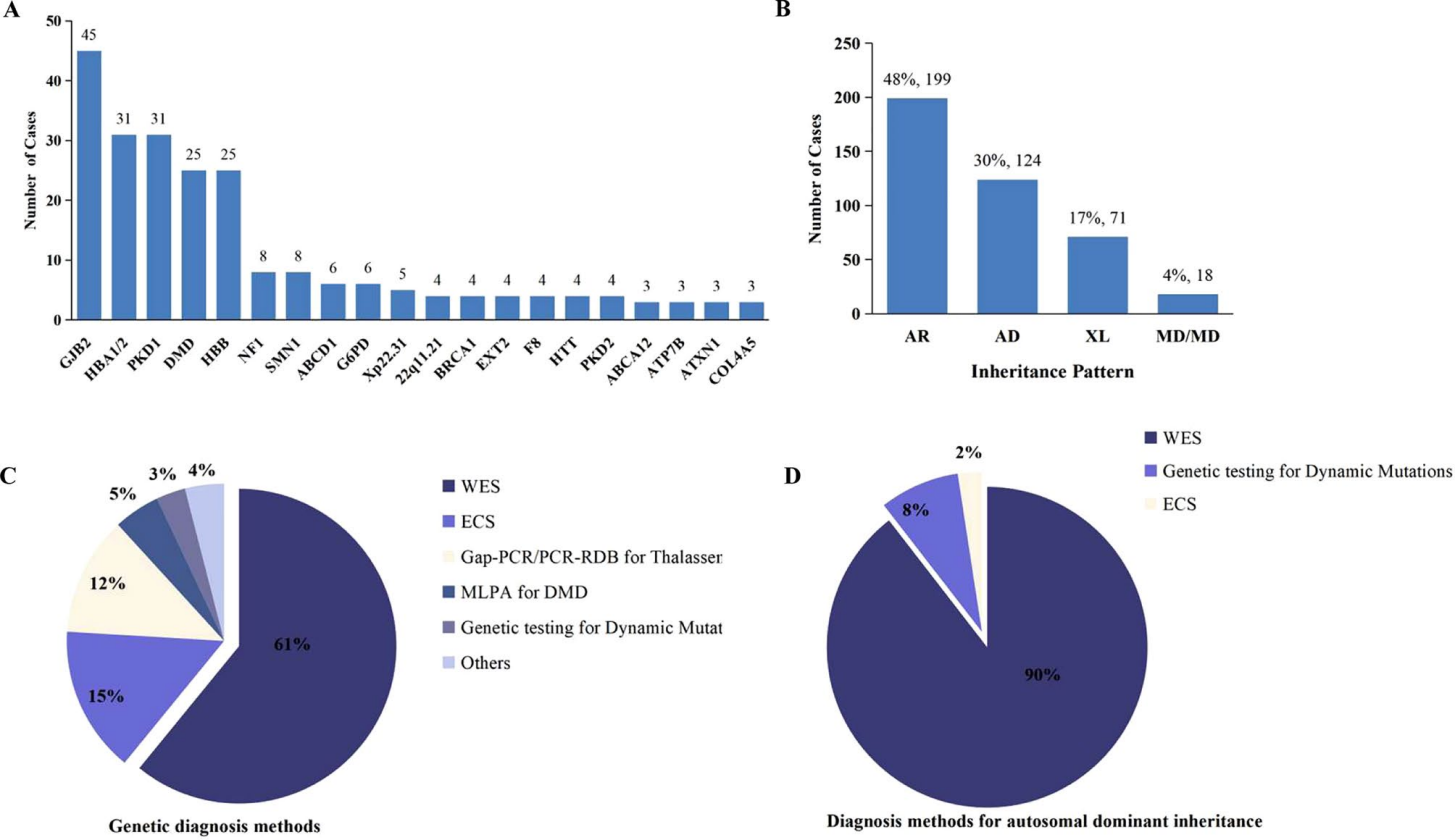
Overall information of PGT-M preclinical work-up. **A**. The top 20 genes most frequently tested in PGT-M cases. The number of detection cases is labeled on the bar chart for each gene. **B**. The cases number and ratio for different inheritance patterns. AD: autosomal dominant; AR: autosomal dominant; XL: X-linked inheritance patterns including XLR, XLD and XL; MD/MD: microdeletion/microduplication. Genetic diagnosis method and the ratio for all cases (**C)** and for autosomal dominant cases (**D**). WES: cases diagnosed by whole-exome sequencing; ECS: cases diagnosed by carrier screening; Cases diagnosed via conventional genetic testing methods for thalassemia gene including Gap-PCR and PCR-RDB, MLPA for Duchenne/Becker muscular dystrophy (DMD/BMD), PCR and capillary electrophoresis for dynamic mutation genes et al. Others represent cases diagnosed by CMA, low-depth high throughput sequencing and so on

A total of 377 families that underwent preclinical work-up for PGT-M using the Asian Screening Array were included in this study, 34 families of which received PGT-M for two genes concurrently. The mean age was 31.4 and 33.2 years for female and male participants, respectively. In total, 150 distinct genes and 8 pathogenic copy number variations (CNVs) were involved in this PGT-M testing (see supplementary materials). The most frequently tested gene within this dataset was *GJB2*, with 45 cases accounting for 10.9% of the total enrolled. Next in frequency were the *HBA1/2* (31 cases), *PKD1* (31 cases), *DMD* (25 cases) and *HBB* (25 cases) genes. Among them, cases of thalassemia, including both α-thalassemia and β-thalassemia, account for as high as 13.6% of the total cases. Other common genes include *NF1*, *SMN1*, *ABCD1*, *G6PD*, *BRCA1*, *EXT2*, *F8*, *HTT* and *PKD2* (Fig. 1A). It is worth noting that the top two CNV Syndromes involved are X-linked ichthyosis (XLI) and DiGeorge syndrome, with deletion in regions of Xp22.31 and 22q11.21, respectively. In addition, cases involving other relatively rare genes or regions also account for nearly half of the total, which indicates that rare genetic disorders constitute an extremely large and clinically important group for PGT-M.

Among all cases, autosomal recessive (AR) inheritance accounts for the largest proportion, reaching 48% (Fig. 1B). Then, cases of autosomal dominant (AD) inheritance account for 30%, with the ratio of male patients to female patients being 1.34:1 (71/53). Cases of X-linked (XL) inheritance include *DMD* (Duchenne Muscular Dystrophy, 25 cases), *ABCD1* (associated with adrenoleukodystrophy, 6 cases), *G6PD* (associated with G6PD deficiency, 6 cases), *F8* (associated with Hemophilia A, 4 cases) and other conditions, most of which are X-linked recessive. In addition, a total of 18 cases with microdeletion/microduplication (MD/MD) was involved in this study to evaluate the feasibility of the PGT strategy based on linkage analysis with the ASA for small CNVs.

### Genetic diagnosis for different inherited disorders

In this study, whole-exome sequencing (WES) confirmed the genetic diagnosis in 243 cases, accounting for approximately 61% of the total cases and 89.5% of those with autosomal dominant inheritance (Fig. 1C and D). Additionally, 15% of at-risk couples (ARCs) are detected when both phenotypically normal partners are found to carry a pathogenic or likely pathogenic (P/LP) variant in the same autosomal recessive gene, or when females carry X-linked variants, through expanded carrier screening (ECS) or carrier screening based on WES. The remaining cases were diagnosed via conventional genetic testing methods, including Gap-PCR/PCR-Reverse Dot Blot (PCR-RDB) for thalassemia (12%), PCR and capillary electrophoresis for dynamic mutation genes testing (3%), multiplex ligation-dependent probe amplification (MLPA) testing for Duchenne/Becker muscular dystrophy (DMD/BMD) (5%) and Spinal Muscular Atrophy (SMA). Moreover, cases involving microdeletions or microduplications were diagnosed via copy number variation (CNV) testing based on Chromosomal Microarray Analysis (CMA) or next-generation sequencing (NGS).

### PGT strategy for different genetic disorders

In our study, we performed linkage analysis using SNPs detected by the ASA for different genetic disorders (Table 1). For most of the cases, the number of SNPs available for linkage analysis in the upstream and downstream regions of target genes/regions is sufficient. However, for a subset of genes such as *HBA1/HBA2*, *NF1*,* SMN1* or genes located on the X chromosome (e.g., *F8*, *DMD*,* ABCD1*), insufficient SNPs or recombination events in the target genomic regions may lead to the failure of linkage analysis. Direct mutation detection for embryos is therefore particularly crucial as a complementary approach to linkage analysis.

**Table 1:** PGT strategy for microdeletions or microduplications and other genetic disorders

Types	Regions/genes	Number of Cases	Related CNV Syndromes/Regions	Sizes/Mutation sites	PGT strategy*
Microdeletion/ Microduplication	Xp22.31	5	X-linked ichthyosis (XLI)	0.79 Mb/1.68 Mb	ASA/ PGT-A
	22q11.21	4	22q11 deletion syndrome (Velocardiofacial/DiGeorge syndrome)	3.15 Mb/2.18 Mb	
	16p11.2	2	16p11.2 recurrent region (proximal, BP4-BP5) (includes TBX6)	0.22 Mb/0.6 Mb	
	17q12	2	17q12 recurrent (RCAD syndrome) region (includes HNF1B)	1.38 Mb/1.48 Mb	
	1q21.1q21.2	2	1q21.1 recurrent region (distal, BP3-BP4) (includes GJA5)	3.54 Mb	
	2q13	1	2q13 recurrent region (proximal) (includes NPHP1)	0.12 Mb	
	Xp22.2p22.12	1	N/A	4.51 Mb	
	Xp22.33	1	Leri-Weill dyschondrostosis (LWD) - SHOX deletion	2.36 Mb	
Trinucleotide repeat expansions	*HTT*	4	Huntington disease	expanded CAG/CGG repeats	ASA/ RP-PCR/ PGT-A
	*ATXN1*	3	Spinocerebellar ataxia 1		
	*ATXN3*	2	Spinocerebellar Ataxia Type 3		
	*FMR1*	2	Fragile X syndrome		
	*ATXN2*	1	Spinocerebellar ataxia 2		
Deletional alpha-thalassemia	*HBA1/2*	33	Thalassemias, alpha-	--^SEA^, -α^3.7^, -α^4.2^	ASA/Gap/ PGT-A
Special genes	*SMN1*	8	Spinal muscular atrophy	Exon 7/8 del	ASA/ PCR-RFLP/ PGT-A

*RP-PCR: repeat-primed PCR and capillary electrophoresis; PCR-RFLP: PCR-restriction fragment length polymorphism

For cases with deletional alpha-thalassemia such as --^SEA^, -α3.7 and -α4.2, gap-PCR analysis for all the embryos was conducted and deletion-carrying embryos can be directly identified via agarose gel electrophoresis (Figure S1 and Figure S2A-B). The gap-PCR results for all the 99 embryos from 20 PGT-M cycles were not contradictory to those of linkage analysis, and 50 embryos were selected as suitable candidates for transfer.

For the *SMN1* gene, no usable SNPs were available within the 1 Mb upstream of the gene for linkage analysis since no probes were designed within this region. Therefore, we additionally employed PCR-restriction fragment length polymorphism (PCR-RFLP) to further confirm whether homozygous deletions of exon 7 or 8 were present in the embryo samples (Figure S3A-B). No cases inconsistent with the linkage analysis results have been identified.

In addition, repeat-primed PCR (RP-PCR) and fragment length analysis was conducted for cases with repeat expansion disorders (related genes like *ATXN1*, *ATXN2*, *ATXN3*, *FMR1*, *HTT*). Combining linkage analysis and direct expansion mutation detection enabled accurate diagnosis of the carrier status of each embryo. Among the cases involving trinucleotide repeat expansions, 24 out of 65 embryos from 12 PGT-M cycles were eligible for transfer.

### PGT strategy and outcomes for de novo pathogenic variant(s)

In conventional PGT-M, linkage analysis typically relies on positive family members. However, for cases involving *de novo* variants or lack of family members, we perform haplotype analysis directly using single spermatozoa, or construct haplotypes for carriers via long-read sequencing (LRS). Here, we enrolled 18 families with different *de novo* variants in 14 genes including *NF1* (neurofibromatosis, 3 cases), *FBN1* (Marfan syndrome, 2 cases), *PKD1* (polycystic kidney disease 1, 2 cases) and others (Table 2). Haplotypes were successfully constructed for all cases, enabling the accurate differentiation between the high-risk haplotype and low-risk haplotype in the affected individual or mutation carriers. However, for the 5 families using the single sperm haplotype methodology, more than 30 single spermatozoa were isolated per family and the single spermatozoa successfully validated for haplotype construction exhibited a relatively low detection rate and accuracy following analysis on the ASA (Figure S4A-B). For the other 13 families, direct haplotype construction was performed for the affected individual using long-read sequencing. Combined with the results of ASA analysis for the couple and embryos, the final SNPs applicable for linkage analysis in PGT-M clinical cycle were identified. Meanwhile, long-read sequencing allows for precise breakpoint detection in deletion cases such as exon46-51 deletion in *DMD* gene and exon3-47 deletion in *FLNA* gene (Figure S5A-B). Targeted Sanger sequencing for directly mutation detection is also essential for the identification of carrier status for embryos. Here, A total of 53 embryos from 12 PGT-M cycles were included in this study. And 18 non-carrier and euploid embryos were selected for transfer.

**Table 2:** PGT strategy for de novo pathogenic variants or lack of family members

Genes	Number of Cases	Related diseases	Mutation sites	PGT strategy*
*NF1*	3	Neurofibromatosis, type 1	c.1062+2T> C	LRS/ASA/Sanger/PGT-A
			c.5380 C> T	SPH/ASA/Sanger/PGT-A
			c.5546G> A(NM_000267.3)	LRS/ASA/Sanger/PGT-A
*FBN1*	2	Marfan syndrome	c.4205G> T	SPH/ASA/Sanger/PGT-A
			c.4930 C> T(NM_000138.5)	LRS/ASA/Sanger/PGT-A
*PKD1*	2	Polycystic kidney disease 1	c.8327_8343dup	SPH/ASA/Sanger/PGT-A
			exon22 del (NM_001009944.3)	LRS/ASA/Gap/PGT-A
*APC*	1	Gastric adenocarcinoma and proximal polyposis of the stomach	c.3497_3501del (NM_000038.6)	LRS/ASA/Sanger/PGT-A
*BCOR*	1	Microphthalmia, syndromic 2	c.1873_1876delinsTTC (NM_017745.6)	LRS/ASA/Sanger/PGT-A
*BRCA2*	1	Breast-ovarian cancer, familial, 2	c.3109 C> T(NM_000059.4)	LRS/ASA/Sanger/PGT-A
*DMD*	1	Duchenne muscular dystrophy	exon46-51 del (NM_004006.2)	LRS/ASA/PGT-A
*EXT1*	1	Exostoses, multiple, type 1	c.797T> C(NM_000127.3)	LRS/ASA/Sanger/PGT-A
*FGFR1*	1	Hartsfield syndrome	c.809G> T(NM_145239.3)	LRS/ASA/Sanger/PGT-A
*FLNA*	1	Otopalatodigital syndrome	exon3-47 del (NM_001456.4)	LRS/ASA/PGT-A
*FOXL2*	1	Blepharophimosis, epicanthus inversus, and ptosis, types 1 and 2	c.843_859dup (NM_023067.4)	SPH/ASA/Sanger/PGT-A
*KRT5*	1	Epidermolysis bullosa simplex	exon7-8 del (NM_000424.4)	SPH/ASA/Sanger/PGT-A
*TP63*	1	Limb-mammary syndrome	c.1986_2028dup (NM_000053.4)	LRS/ASA/Sanger/PGT-A
*TSC2*	1	Tuberous sclerosis-2	c.3377del (NM_000548.5)	LRS/ASA/Sanger/PGT-A

*LRS: long-read sequencing; ASA: the Asian Screening array; Sanger: targeted Sanger sequencing; SPH: Single sperm haplotyping; Gap: Gap-PCR

### PGT strategy and outcomes for microdeletions or microduplications

**Fig. 2:**
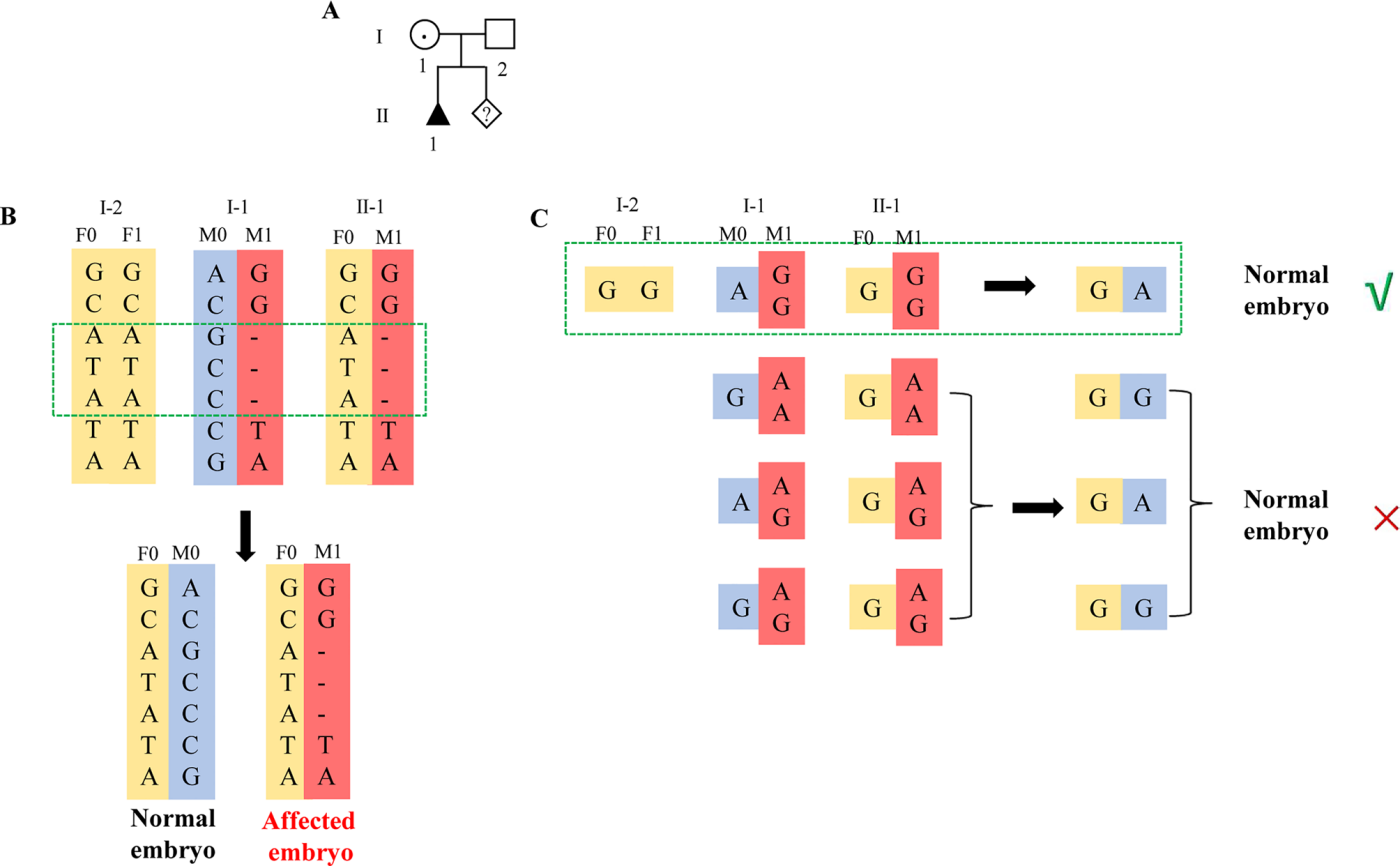
Haplotyping analysis for microdeletions (B) and tandem microduplications (C). **A**. Pedigree chat with microdeletions or tandem microduplications in Mother (І-1) and proband (Ⅱ-1). **B**. F0/F1 represents the two haplotypes for healthy spouse; M0 and M1 represents the low-risk and high-risk haplotypes for the carrier respectively. The SNPs in the green dashed box represented the informative SNPs within the deletion region for embryo haplotyping analysis. **C**. The chart depicts the distribution pattern of heterozygous SNP (A/G detected by ASA) in carriers located within the duplication region. Only the homozygous SNPs in the unaffected spouse and affected proband within duplication region can be selected for linkage analysis (green dashed box)

Due to the limited resolution of conventional PGT for aneuploidy or structural rearrangement (PGT-A/SR) (typically detects > 4 Mb fragments), the PGT strategy based on SNP haplotyping is more applicable for prospective parent with small pathogenic copy number variants. Here, 18 families with microdeletions or microduplications (< 4 Mb) were performed SNP haplotyping analysis based on the Asian Screening Array (Table 1). Informative SNPs flanking or within the target region were selected for linkage analysis to establish haplotypes of prospective parents. Notably, within the deletion region, SNPs that are inconsistent with the carrier and homozygous in the unaffected spouse can be regarded as informative markers (Fig. 2A-B). And for tandem duplications, the homozygous SNPs in the unaffected spouse and affected proband within duplication region can be selected for linkage analysis (Fig. 2C). In PGT clinical work-up, those informative SNPs detected by the ASA can assist in distinguishing the carrier status of embryos. A total of 23 embryo testing cycles were performed for those families with microdeletions or microduplications. After undergoing linkage analysis and CNV detection, 30 embryos out of 98 were ultimately deemed suitable for transfer. Successful pregnancies have been achieved in 8 families as of July 2025.

### Overall chromosomal abnormalities in PGT-M and pregnancy outcome

**Fig. 3:**
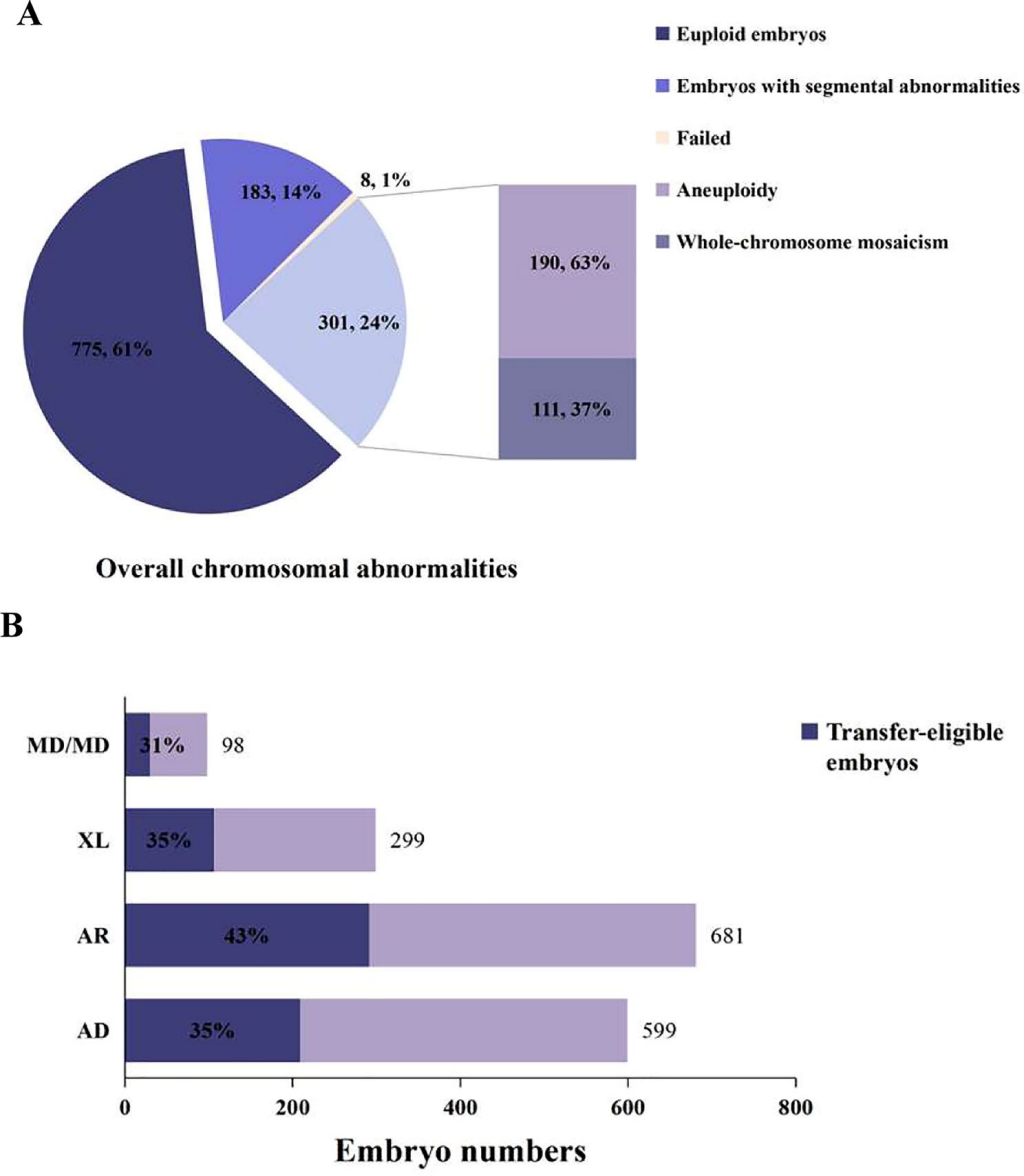
Overall chromosomal abnormalities in PGT-M clinical work-up (A) and transfer-eligible embryos for different inheritance patterns (B). **A**. Euploid embryos accounted for 76% of the total. Aneuploid embryos accounted for 24% of the total, of which aneuploid embryos and embryos with whole-chromosome mosaicism accounted for 63% and 37%, respectively. Those with only chromosomal segment abnormalities (including segmental mosaicism) accounted for 14.21%. The additional 1% of embryos represented test-failed embryos. **B**. The whole obtained embryos (numbers are labeled next to the bars) and transfer-eligible embryos (dark purple) for different inheritance patterns

Overall, 1677 embryos obtained from 367 PGT-M cycles were included in this study, with an average of 4–5 embryos retrieved per cycle. A total of 1267 embryos underwent chromosomal abnormality detection. Among these embryos, euploid embryos accounted for 61.16% (775/1267), while embryos with aneuploidy (including whole-chromosome mosaicism) accounted for 23.76% (301/1267), and those with only chromosomal segment abnormalities (including segmental mosaicism) accounted for 14.21% (183/1267) (Fig. 3A). When analyzing the subset of embryos with numerical chromosomal abnormalities, we found that roughly 37% of them were mosaic for whole chromosomes (111/301). The additional 1% of embryos represented test-failed embryos, which might be attributed to biopsy failure, amplification failure, or detection failure. Additionally, the transfer-eligible embryo rates were 35%, 45%, and 35% for autosomal dominant (AD), autosomal recessive (AR), and X-linked (XL) inheritance patterns, respectively, following linkage analysis and chromosomal abnormality testing (Fig. 3B). A total of 188 PGT cycles resulted in clinical pregnancy, among which 120 healthy live birth were delivered as of March 2026. Among cases with successful pregnancies and completed prenatal diagnosis (chorionic villus sampling or amniocentesis), the consistency rate between PGT results and prenatal validation results was 100%.

## Discussion

Although blastocyst trophectoderm biopsy is currently adopted in most PGT-M clinical application, there remains a risk of allele drop-out (ADO) in direct locus-specific testing due to the limited DNA quantity [30, 31]. Therefore, indirect haplotype analysis is indispensable for the accurate diagnosis of embryos [4, 32]. Genome-wide SNP array- or NGS-based haplotyping strategies have demonstrated more advantages, such as cost-effectiveness, high throughput, and a greater number of informative SNPs, over conventional targeted amplification strategies [10]. We also demonstrated the applicability of the ASA in the Chinese population for PGT-M in our previous study [9]. Here, we have summarized the PGT-M cases based on the ASA over the past three years, proposed and analyzed the clinical utility of PGT-M strategies tailored to different genetic disorders, thereby providing insights for a broader range of clinical scenarios.

In recent years, PGT technology has brought new hope to families affected by genetic or chromosomal disorders, particularly those carrying rare monogenic mutations or complex chromosomal rearrangements [33]. Couples affected by monogenic diseases have shown growing willingness to utilize PGT-M for assisted reproduction, following rigorous ethical review and comprehensive genetic counseling. With the widespread application of clinical genetic testing methods such as WES and ECS, an increasing number of rare genetic diseases have been diagnosed [34, 35]. In our study, genetic diagnoses in more than 76% of cases were confirmed by WES and ECS, highlighting the clinical utility of WES in the diagnosis of genetic disorders and ECS in identifying potential genetic disorders.

Regarding the amplification method for TE cells, typically, 5–10 TE cells were subjected to whole-genome amplification (WGA) via either MDA or MALBAC, depending on the mutation type and the complexity of the gene locus [36, 37]. Our experience indicates that both methods exhibited comparable performance in detecting SNVs or small indels for most genes. While, MDA can significantly improve the amplification success rate of regions with high GC content and high-repeat sequences. Therefore, for mutations in *PKD1*, *HBA1/2*, *SMN1* and trinucleotide repeat genes, we consistently select MDA for amplification with high amplification yield and long fragment output. For other genetic conditions, during the PGT-M preclinical stage, we first evaluated the efficiency of MALBAC in amplifying and mutations detecting on oral mucosal cells. If MALBAC exhibits insufficient efficiency, MDA amplification is an alternative option, thereby finalizing the amplification strategy for TE cells. Among the embryos included in this study, a total of 1100 embryos were amplified using MALBAC, while the remaining 577 embryos were amplified using MDA. And no significant variation was observed in the detection performance of the ASA for MDA versus MALBAC amplification products.

Following whole-genome amplification (WGA), the amplified samples underwent both direct locus-specific testing and linkage analysis simultaneously. In our study, we summarized several distinct locus-specific detection methods for different genetic conditions. For SNVs or small indels, target-specific amplification and Sanger sequencing was applied for direct mutation detection of the embryos. When combined with linkage analysis results, ADO still occurs occasionally in direct mutation detection. Specifically, the ADO rate of MALBAC method was 1.36% (15/1100), whereas that of MDA amplification was significantly lower at only 0.35% (2/577), which mainly attributed to their distinct amplification mechanisms. The *SMN1* gene represents another special case. Although it cannot distinguish between normal embryos and carrier embryos, PCR-RFLP still serves as a reliable supplementary method for identifying embryos with *SMN1* homozygous deletion when valid SNPs are lacking on the ASA. For deletional alpha-thalassemia or large deletion mutations with known breakpoints, gap-PCR combined with agarose gel electrophoresis enable the identification of embryos with homozygous deletion. By employing these distinct mutation validation methods combined with linkage analysis, the two approaches cross-validate each other to ensure the accuracy of embryo detection results.

The Asian Screening Array, which harbors approximately 700,000 SNP loci specific to East and Southeast Asian populations, offers a scalable and cost-effective approach for linkage analysis of most genetic variants [38]. In our study, ASA provided a robust solution for linkage analysis across more than 150 genes and multiple microdeletion regions involved in the research. A sufficient number of valid SNPs were present both upstream, downstream, and within the genes, ensuring the accuracy of linkage analysis, except for genes with special genomic locations, such as *SMN1*,* F8*. We also summarized specialized linkage analysis methodologies for *de novo* mutations based on single sperm or long-read sequencing. However, the single sperm haplotype methodology is usually labor and cost consuming because of the high technical demand for single sperm selection and validation. Long-read sequencing offers a more optimal strategy attributed to its high accuracy and efficiency in PGT-M haplotype phasing involving *de novo* mutations. Notably, long-read sequencing allows for precise breakpoint detection in deletion cases, which in turn supports direct mutation detection for embryos. Meanwhile, linkage analysis based on informative SNPs flanking or within the target regions is an effective PGT strategy for couples with small CNVs, including tandem duplications [39, 40].

Overall, the PGT methods based on ASA established in this study provides an efficient and feasible technical solution for the clinical application of PGT under different disease types and genetic backgrounds. Although, in comparison with many currently developed comprehensive PGT methods [41–43], the SNP array-based linkage analysis protocol requires an additional detection of chromosomal numerical or structural abnormalities. Nonetheless, this method is characterized by its distinct advantages such as high specificity for monogenic disorder screening, stable technical performance, and cost-effectiveness. In addition, for embryos in which the pathogenic variants have been clearly identified via linkage analysis or direct mutation testing, patients can choose to skip the detection of chromosomal anomalies, thereby reducing the overall testing costs.

## Conclusion

In conclusion, based on comprehensive PGT-M datasets accumulated over three years in our center (involving 377 pedigrees and 1677 embryos), our findings systematically illustrate the performance of the ASA-based PGT workflow that synergizes linkage analysis, direct mutation testing, and chromosomal CNV screening. For diverse genetic conditions, we developed customized PGT testing strategies and assessed their clinical performance. Our results provided data support and practical references for the individualized clinical application of PGT-M technology.

## Electronic Supplementary Material

Below is the link to the electronic supplementary material.

Supplementary Material 1: Figure S1. Detailed primer design for Gap-PCR in HBA1/2. The simplified diagram for α-globin gene cluster and the location for represented deletions. The rough location of 8 pairs of Gap-PCR primers (P1-P8) are marked in the diagram. The P1 product represents –^SEA^; The P6 product represents –α^3.7^; The P7 product represents –^4.2^; Primers P2 and P3 amplified bands located within the SEA deletion region; Primers P4 and P5 amplified bands located within the α^3.7^ deletion region; Primers P8 amplified bands located within the α^4.2^ deletion region.

Supplementary Material 2: Figure S2. Gap-PCR (A) and the ASA haplotyping results (B) for a family with deletional alpha-thalassemia of –SEA and-α3.7. **A.** Agarose gel electrophoresis for the Gap-PCR products for the parents and 7 embryos. F, M, E1 represents father, mother and the embryos, respectively. The phenotype of Mother and E1-6 is --^SEA^/-α^3.7^; Father and E1-1 is --^SEA^/αα; E1-2 and E1-4 is -α^3.7^/αα; E1-3, E1-5 and E1-7 is --^SEA^/--^SEA^. **B**. The light blue frame with slashes refers to the --^SEA^ haplotype of Father. And the dark blue frame refers to the normal haplotype. The light orange frame with slashes refers to the --^SEA^ haplotype of Mother; The dark orange frame with slashes refers to the -α^3.7^ haplotype of Mother.

Supplementary Material 3: Figure S3. PCR-RFLP (A) and the ASA haplotyping results (B) for a family with exon 7 or 8 deletion in SMN1. **A.** Agarose gel electrophoresis for the G1/G2 products after restriction enzyme digestion. For G1 product, the carrier and normal one has the band of 188 bp and/or 164 bp, while homozygous deletion of exon 7 of the SMN1 gene only have the band of 164 bp. For G2 product, the carrier and normal one has the band of 188 bp and/or 120 bp, while homozygous deletion of exon 8 of the SMN1 gene only have the band of 120 bp. **B**. The light blue frame with slashes refers to the high-risk haplotype of Father. And the dark blue frame refers to the low-risk haplotype. The light orange frame with slashes refers to the high-risk haplotype of Mother; The dark orange frame with slashes refers to the low-risk haplotype of Mother.

Supplementary Material 4: Figure S4. Singer sperm haplotyping results for male with c.4205G > T in FBN1 gene (A) and c.843_859dup in FOXL2 gene (B). The light blue frame with slashes refers to the high-risk haplotype of the male deduced by single sperm. And the dark blue frame refers to the low-risk haplotype. F, M and spm represent father, mother and singer sperm; The detected SNPs (A/T/C/G) are marked next to the frame, of which ‘0’ represents the loci with failed detection.

Supplementary Material 5: Figure S5. Precise breakpoint detection by long read sequencing for female with exon46-51 deletion in DMD gene (A) and exon3-47 deletion in FLNA gene (B). **A**. The IGV shows the breakpoint of chrX: 31,765,600 and chrX:31,954,240 for *DMD* gene. **B**. The IGV shows the breakpoint of chrX: 153,596,768 and chrX:153,573,912 for *FLNA* gene.

Supplementary Material 6

## Data Availability

The raw data supporting the conclusions of this article will be made available by the authors, without undue reservation.
